# Supplementary figures and images for: Bupivacaine as a euthanasia agent for African Clawed Frogs (Xenopus laevis)
Source: PLoS One. 2022 Dec 21;17(12):e0279331. doi: 10.1371/journal.pone.0279331 (PMC9770357; doi:10.1371/journal.pone.0279331)

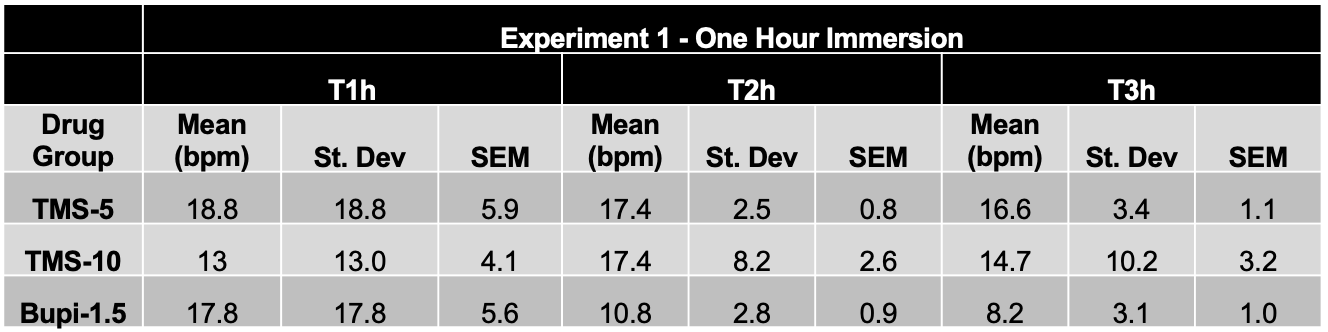

Supplement: S1 Table — (TIF) [file pone.0279331.s001.tif]

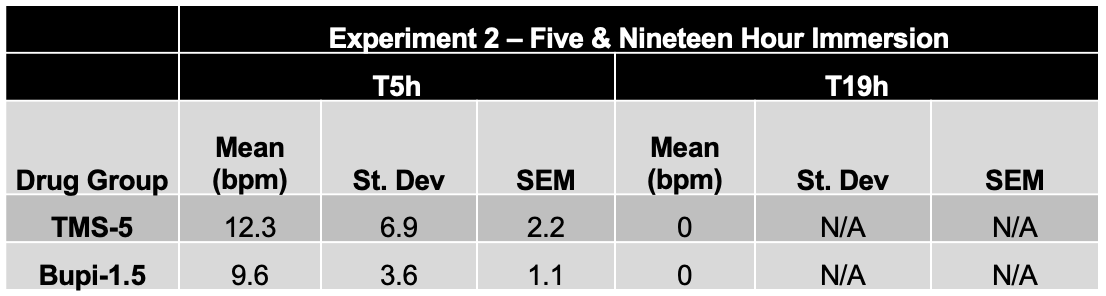

Supplement: S2 Table — (TIF) [file pone.0279331.s002.tif]
