# Supplementary material for: Bupivacaine as a euthanasia agent for African Clawed Frogs (Xenopus laevis)
Source: PLoS One. 2022 Dec 21;17(12):e0279331. doi: 10.1371/journal.pone.0279331 (PMC9770357; doi:10.1371/journal.pone.0279331)
Supplement: Attachment — Submitted filename: Response to Reviewers.docx [file pone.0279331.s003.docx]

Dear Reviewers,

Thank you again for your effort in reviewing our manuscript. Please find addressed to the journal requirements and your comments addressed below.

Sincerely,

Kaela Navarro

______________________________________________________________________________

When submitting your revision, we need you to address these additional requirements.

*1. Please ensure that your manuscript meets PLOS ONE's style requirements, including those for file naming. The PLOS ONE style templates can be found at* 
[*https://journals.plos.org/plosone/s/file?id=wjVg/PLOSOne_formatting_sample_main_body.pdf*](https://journals.plos.org/plosone/s/file?id=wjVg/PLOSOne_formatting_sample_main_body.pdf) *and* 
[*https://journals.plos.org/plosone/s/file?id=ba62/PLOSOne_formatting_sample_title_authors_affiliations.pdf*](https://journals.plos.org/plosone/s/file?id=ba62/PLOSOne_formatting_sample_title_authors_affiliations.pdf)

Response: This has been revised.

*2. Please include your full ethics statement in the ‘Methods’ section of your manuscript file. In your statement, please include the full name of the IRB or ethics committee who approved or waived your study, as well as whether or not you obtained informed written or verbal consent. If consent was waived for your study, please include this information in your statement as well.*

Response: Thank you for the comment; this was included in our manuscript and can be located in the revised version in lines 104-108. The additional requirements of anesthesia and euthanasia can be found within the methods section.

*3. As part of your revision, please complete and submit a copy of the Full ARRIVE 2.0 Guidelines checklist, a document that aims to improve experimental reporting and reproducibility of animal studies for purposes of post-publication data analysis and reproducibility:* [*https://arriveguidelines.org/sites/arrive/files/Author%20Checklist%20-%20Full.pdf*](https://arriveguidelines.org/sites/arrive/files/Author%20Checklist%20-%20Full.pdf) *(PDF). Please include your completed checklist as a Supporting Information file. Note that if your paper is accepted for publication, this checklist will be published as part of your article.*

Response: This checklist has been completed and uploaded as a supplemental file.

*4. We note that the grant information you provided in the ‘Funding Information’ and ‘Financial Disclosure’ sections do not match.*

*When you resubmit, please ensure that you provide the correct grant numbers for the awards you received for your study in the ‘Funding Information’ section.*

Response: This has been clarified in the resubmission process.

*5. Thank you for stating the following in the Acknowledgments Section of your manuscript: 
   "We would like to thank Andrea Craig and Alberto Gaudiel for their assistance in care for the animals. The study was supported by the Stanford Department of Comparative Medicine."*

*We note that you have provided funding information that is not currently declared in your Funding Statement. However, funding information should not appear in the Acknowledgments section or other areas of your manuscript. We will only publish funding information present in the Funding Statement section of the online submission form. 
Please remove any funding-related text from the manuscript and let us know how you would like to update your Funding Statement. Currently, your Funding Statement reads as follows: 
 "This work was supported by Stanford's Department of Comparative Medicine. The funders had no role in study design, data collection and analysis, decision to publish, or preparation of the manuscript. The content is solely the responsibility of the authors and does not necessarily represent the official views of the Stanford Department of Comparative Medicine or any other agency of the State of California."*

*Please include your amended statements within your cover letter; we will change the online submission form on your behalf.*

Response: Thank you. The funding statement in the manuscript has been removed. We have addressed the statement in our cover letter.

*6. We note that you have indicated that data from this study are available upon request. PLOS only allows data to be available upon request if there are legal or ethical restrictions on sharing data publicly. For more information on unacceptable data access restrictions, please see* [*http://journals.plos.org/plosone/s/data-availability#loc-unacceptable-data-access-restrictions*](http://journals.plos.org/plosone/s/data-availability#loc-unacceptable-data-access-restrictions)*.*

Response: We have uploaded supplementary files to provide a minimal data set. All other data are included within the manuscript.

3. Have the authors made all data underlying the findings in their manuscript fully available?

The [PLOS Data policy](http://www.plosone.org/static/policies.action#sharing) requires authors to make all data underlying the findings described in their manuscript fully available without restriction, with rare exception (please refer to the Data Availability Statement in the manuscript PDF file). The data should be provided as part of the manuscript or its supporting information, or deposited to a public repository. For example, in addition to summary statistics, the data points behind means, medians and variance measures should be available. If there are restrictions on publicly sharing data—e.g. participant privacy or use of data from a third party—those must be specified.

Reviewer #1: No

Reviewer #2: No

Response: We have uploaded supplementary files to provide a minimal data set. All other necessary data are included within the manuscript.

5. Review Comments to the Author

Please use the space provided to explain your answers to the questions above. You may also include additional comments for the author, including concerns about dual publication, research ethics, or publication ethics. (Please upload your review as an attachment if it exceeds 20,000 characters)

**Reviewer #1*:*** *Dear Author, This is interesting. However, please elaborate the practical utility and implication of the present and advantage over other existing models.
How these data can be used to expand knowledge in clinical or basic research files.*

**Response:** Thank you for your comment. We have added additional information elaborating on the importance of *Xenopus* as an animal research model, why large group culls may be necessary, and the importance of alternative euthanasia agents in the introduction.

**Reviewer #2:** *The author is willing to share raw data upon request, however states that restrictions will apply as far as posting to a public repository. It is not included with the manuscript*.

**Response:** We have uploaded supplementary files to provide a minimal data set. All other necessary data are included within the manuscript.

Manuscript review PONE-D-22-17202, entitled "Bupivacaine as a euthanasia agent for African Clawed Frogs (Xenopus laevis)".
Overall Comments: This manuscript describes the immersion of African Clawed frogs in bupivacaine or TMS for humane euthanasia. This information is beneficial to the research community and describes a potential novel euthanasia agent for this species, in that bupivacaine successfully lowered the heart rate more quickly than the standard immersion agent and produced irreversible death. This give the community another, safer, agent to use. It also shows the importance of using a secondary method of euthanasia when following the AVMA Guidelines for the use of TMS in this species. The study is described well and conclusion are appropriate.

**Reviewer 2:** *Abstract, Line 31- 9 hours is stated, this should be 19.*
**Response:** Thank you, this has been revised.

**Reviewer 2:** *Introduction, Line 47- Please add the word “and” between endocrinology and toxicology.*
**Response:** Thank you, this has been revised.

**Reviewer 2:** *Materials and Methods, Line 110- Please add a sentence prior to “A celiotomy was performed…” on how it was confirmed a surgical plane of anesthesia was attained.*
**Response:** This has been revised to confirm a surgical plane of anesthesia had been attained prior to celiotomy and how it was performed.
**Reviewer 2:** *Results, Line 139- Please add a reference to Figure 2.*
**Response:** This has been added.

**Reviewer 2:** *Discussion, Line 228-9- “brain death or irreversible” Please complete this sentence.*
**Response:** Thank you, this has been revised.

**Reviewer 2:** *Figure 1B. There should be an indication that frogs without a heart beat had a celiotomy performed at the 5h timepoint.*

**Response:** The figure has been updated to reflect the celiotomy at 5h and the figure text has been updated to reflect when celiotomies were performed and for which animals.
